# Hypermethylation of the tumor-suppressor cell adhesion molecule 1 in human papillomavirus-transformed cervical carcinoma cells

**DOI:** 10.3892/ijo.2015.2945

**Published:** 2015-04-01

**Authors:** HYUN JU WOO, SUNG JIN KIM, KYUNG-JOO SONG, SUNG SOON KIM, CHEOL-HEE YOON, BYEONG-SUN CHOI, JEE EUN RHEE

**Affiliations:** Division of AIDS, Center for Immunology and Pathology, Korea National Institute of Health, Chungbuk, Republic of Korea

**Keywords:** human papillomavirus, CADM1, hypermethylation, cervical cancer

## Abstract

Epigenetic modification at CpG islands located on the promoter regions of tumor-suppressor genes has been associated with tumor development in many human cancers. Our study showed that the cell adhesion molecule 1 (CADM1) is downregulated in human papillomavirus (HPV)-infected cervical cancer cell lines via its hypermethylation and demethylation using 5-aza-2′-deoxycyticine (5-aza-dC) restored the expression of CADM1 protein. Overexpression of CADM1 inhibited cell proliferation. p53 was involved in the regulation of CADM1. Our results demonstrate that epigenetic alteration of CADM1 was more frequent in HPV-positive cervical cancers and that restoration of CADM1 expression may be a potential strategy for cervical cancer therapy.

## Introduction

Cervical cancer is one of the most common malignancies affecting women worldwide, and it is estimated that cervical carcinoma is responsible for 274,000 deaths annually (1). It is well established that infection of high-risk human papillomavirus (hr-HPV) is necessary for cervical cancer development, and hr-HPV DNA can be detected in almost all cervical carcinomas (2). Carcinogenesis by hr-HPV relies primarily on the expression of two virally encoded oncoproteins, E6 and E7 (3). These act synergistically to immortalize and transform the infected cells, partly via their ability to degrade p53 and Rb, respectively (4). p53 is a tumor-suppressor protein with a sequence-specific DNA-binding domain that plays an important role in transcriptional regulation (5,6). This protein acts via a variety of mechanisms, including cell-cycle arrest, induction of apoptosis and cellular senescence (6). Loss of normal p53 function occurs in a significant proportion of human tumors and primarily induces abnormal expression of many target genes (6,7). Noteworthy, this abnormal expression of several p53 target genes is caused by DNA methylation (8–10). Together with genetic factors, epigenetic factors have been suggested as contributing mechanisms in cervical carcinogenesis (11,12). Epigenetic modifications, particularly DNA methylation in promoter regions, are recognized as common molecular alterations in tumor cells and act via the complete blockage of transcription of tumor-suppressor genes (13,14). Previous data related to cervical cancer showed that *DAPK1, FHIT, MGMT, CDKN2A, CADM1* and *MAL* were frequently methylated genes in cervical carcinogenesis (12,15).

The cell adhesion molecule 1 *(CADM1)* gene encodes a member of the immunoglobulin superfamily and is one of the crucial tumor suppressors involved in cell adhesion. It is also known as *TSLC1, Necl-2, IgSF4A* and *SynCAM1* (16). The *CADM1* gene is frequently down regulated epigenetically in a variety of advanced-stage human cancers of the lung, prostate, liver, pancreas, and breast (16,17). Reduced CADM1 expression disrupts cell-cell adhesion in epithelial cells and triggers tumor cell invasion and metastasis (17).

In addition to the epidemiological studies of CADM1 in cervical cancer performed to date, the functional involvement of CADM1 in tumor suppression has been reported by very few studies and remains unclear (18,19). In this study, we explored the relationship between CADM1 methylation status and its expression in various cervical cancer cell lines. Concomitantly, we investigated whether CADM1 expression could be restored in cervical cancer cell lines expressing methylated CADM1 that were treated with the demethylation reagent 5-aza-2′-deoxycytidine (5-aza-dC). In addition, we determined the effect of CADM1 overexpression on cell proliferation, and the role of p53 in the regulation of CADM1 expression in cervical cancer cell lines.

## Materials and methods

### Cell culture

The human embryonic kidney (HEK) 293T and cervical cancer cells (C33A, HeLa, SiHa and CaSki) used in this study were purchased from ATCC (Rockville, MD, USA). The cells were cultured in Dulbecco’s modified Eagle’s medium (DMEM) supplemented with 10% fetal bovine serum at 37°C in a humidified atmosphere with 5% CO_2_. The media used in this study contained 100 U/ml of penicillin and 100 μg/ml of streptomycin (Invitrogen, Carlsbad, CA, USA).

### Kits, reagents and antibodies

5-Aza-2′-deoxycytidine (5-aza-dC) and 5-Fluorouracil (5-FU) were purchased from Sigma Chemical Co. (St. Louis, MO, USA). The Cell Count Kit-8 (CCK-8) was obtained from Dojindo Molecular Technology (Tokyo, Japan). The TRIzol was purchased from Invitrogen. The ECL western blotting kit was obtained from Amersham (Arlington Heights, IL, USA), and Immobilon-P membranes were obtained from Millipore Corp. (Bedford, MA, USA). Anti-p53 and anti-β-actin antibodies were purchased from Santa Cruz Biotechnology (Santa Cruz, CA, USA), the anti-CADM1 antibody was obtained from Abnova (Walnut, CA, USA), the anti-phospho-p53 antibody was obtained from Cell Signaling Technology (Danvers, MA, USA), and horseradish peroxidase (HRP)-conjugated anti-mouse IgG and anti-rabbit IgG were obtained from Santa Cruz Biotechnology.

### qRT-PCR

Total RNA was extracted from cells using the TRIzol reagent (Invitrogen) according to the manufacturer’s instructions, and 2 μg of total RNA was transcribed using the GoScript™ Reverse Transcription System (Promega, Madison, WI, USA) and random primers, according to the manufacturer’s instructions. Quantitative real-time PCR analysis was performed on a StepOnePlus™ Real-time PCR system (Applied Biosystems, Foster City, CA, USA) with SYBR Green. The primer sequences for CADM1 were 5′-CCACAGGTGATGGGCAGAA-3′ (forward), 5′-TCGCAACCTCTCCCTCGAT-3′ (reverse). The primer sequences for β-actin were 5′-ATGCTTCTAGGCGGACTATGA-3′ (forward), 5′-TTTCTGCGCAAGTTAGGTTTT-3′ (reverse). The expression of CADM1 relative to that of β-actin in each sample was calculated and compared.

### Preparation of cell lysates and western blot analysis

Cell lysates were prepared by suspending various cervical carcinoma cell lines in 1X RIPA lysis buffer (Invitrogen) supplemented with a protease inhibitor cocktail (Roche Diagnostics, Indianapolis, IN, USA). The quantitation of proteins was performed using a Micro BCA kit (Pierce, Rockford, IL, USA). Equivalent amounts of protein lysates (20 μg) were electrophoresed on 10% Tris-glycine gel with Tris/glycine/SDS buffer. The proteins were electrotransferred onto Immobilon-P membranes, which were incubated overnight with primary antibodies raised against CADM1 (Abnova), p53 (Santa Cruz Biotechnology), phospho-p53 (Cell Signaling), and β-actin (Santa Cruz Biotechnology) at 4°C. Membranes were then washed with Tris-buffered saline (TBS) containing 0.1% Tween-20 (TBST) and incubated with the appropriate secondary antibodies for 1 h. The detection of each protein was performed using the ECL western blotting kit according to the manufacturer’s instructions. Densitometry was carried out using ImageQuant TL software (Amersham). Arbitrary densitometric units of the protein of interest were corrected using the densitometric units of β-actin.

### 5-Aza-2′-deoxycytidine (5-aza-dC) treatment

5-Aza-dC was dissolved in dimethylsulfoxide (DMSO) and stored at temperatures below −20°C. Final 5-aza-dC concentrations (1–100 μM) were prepared by adding an appropriate amount of the stock solution directly to the culture medium. To identify whether cells were restored after treatment with 5-aza-dC, cells were treated with the drug for 3 days, and the media containing 5-aza-dC was changed every 24 h. Cells were used for the cell proliferation assay or western blot analysis.

### Cell proliferation assay

Cell proliferation was determined using the Cell Count Kit-8 (CCK-8) assay, which reflects cell viability, as described (20). Briefly, cervical cancer cells (C33A, HeLa, SiHa and CaSki) were cultured overnight in 96-well plates. When cells reached a confluency of approximately 70%, they were treated with 5-aza-dC (0–100 μM) for 3 days. Subsequently, 10 μl of the CCK-8 solution was added to each well of the plates. After incubation for 4 h, absorbance was measured at 450 nm using a multi-well plate reader. All assays were performed in triplicate.

### Pyrosequencing

CpG island DNA methylation status was determined by sequencing bisulfite-modified genomic DNA. Briefly, genomic DNA samples were extracted from various cervical cancer cells using the QIAamp DNA Mini kit (Qiagen, Valencia, CA, USA). Genomic DNA (200 ng) was converted with the EZ DNA Methylation kit (Zymo Research, Orange, CA, USA), according to the manufacturer’s protocol. The primers used in pyrosequencing assays [sense, 5′-TTGTTTTGTTAATTAGGGGATTTG-3′; and antisense, 5′-(biotin) CACACCCAATACATCTAACCTA-3′] were used for PCR amplification of the CADM1 promoter region (nucleotides -444 to -305). The size (140 bp) and purity of each amplicon were confirmed by agarose gel electrophoresis. Quantitative pyrosequencing analyses were performed using the PyroMarkQ96 ID system (Qiagen), according to the protocol provided by the manufacturer. The results were analyzed using PyroMark Q96 ID software 2.5 (Qiagen).

### 5-Fluorouracil (5-FU) treatment

Stock solutions of this compound were prepared in dimethylsulfoxide (DMSO) and stored at temperatures below −20°C. Further dilutions were made in DMEM before use. C33A and SiHa cells were plated in six-well plates and cultured. Twenty-four hours after plating, cells were incubated with 5-FU (50 μM) for indicated times from 0 h to 24 h. The cells were used to detect CADM1, phospho-p53, p53 and β-actin using western blot analysis.

## Results

### CADM1 expression is reduced in HPV-infected cervical cancer cells

**Figure 1:**
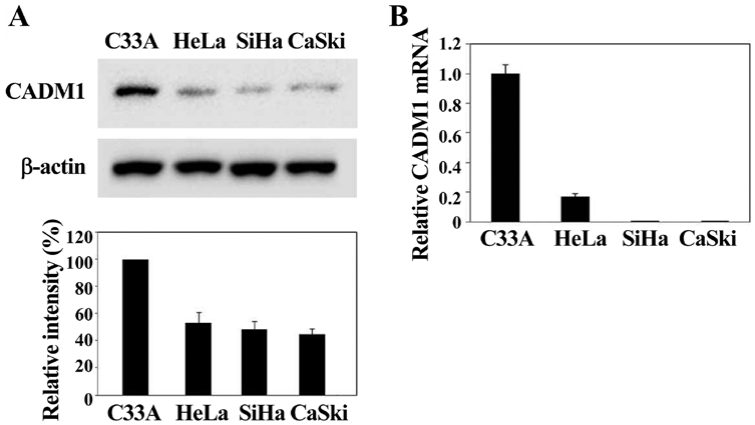
CADM1 expression was downregulated in cervical cancer cell lines. The CADM1 protein (A) and mRNA (B) levels were detected using western blotting and quantitative real-time PCR, respectively, in three HPV-positive cervical cancer cell lines (HeLa, SiHa and CaSki) and one HPV-negative cancer cell line (C33A). Actin was used as a loading control. The relative intensity of CADM1 proteins was determined from scanned western blots using ImageQuant, and the signals detected for the C33A cell line were set as 100%.

To explore whether HPV infection is related to CADM1 expression, we investigated the level of CADM1 in various cervical cancer cells, such as C33A (HPV-negative), HeLa (HPV18-positive), SiHa and CaSki (HPV16-positive) cells. Western blot analyses revealed that the CADM1 protein was predominantly expressed in HPV-negative C33A and decreased in HPV-positive HeLa, SiHa and CaSki cells (Fig. 1A). In addition, we measured CADM1 mRNA levels in all cell lines using real-time quantitative RT-PCR and compared them with the average CADM1 mRNA level. The CADM1 mRNA was mainly expressed in HPV-negative cervical cells. In contrast, the CADM1 mRNA was undetectable in three cervical carcinoma cell lines (i.e., HeLa, SiHa, and CaSki) (Fig. 1B). Thus, we observed either a complete loss of, or a marked decrease in CADM1 mRNA expression in the HPV-infected cervical cancer cell lines analyzed. *CADM1* gene silencing was significantly observed in HPV-induced cervical carcinoma cell lines. These data suggest that CADM1 silencing is a critical requirement for the development of HPV-induced cervical carcinogenesis.

### Decreased expression of CADM1 is caused by hypermethylation of the CADM1 promoter

**Figure 2:**
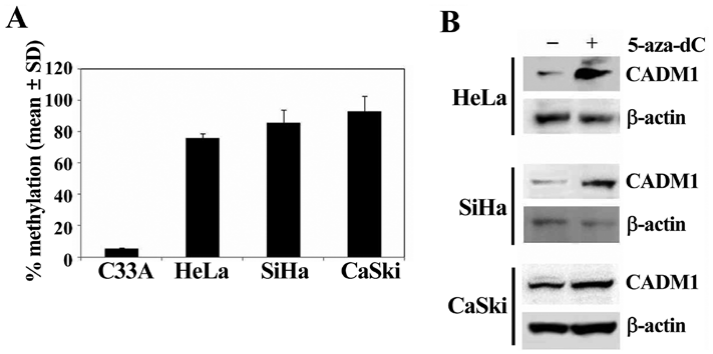
DNA hypermethylation underlies *CADM1* silencing. (A) The methylation status of the *CADM1* gene was detected by pyrosequencing in cervical cancer cell lines. The mean levels of DNA methylation on the *CADM1* promoter region are shown for each cell line. (B) Western blot assay showed that CADM1 proteins that were silenced by the methylation of the corresponding gene and were upregulated after treatment with 10 μM 5-aza-dC for 72 h compared with no treatment with the demethylating agent.

We examined the DNA methylation status of the promoter region of CADM1 (nucleotides -444 to -305) to gain insight into the contribution of gene promoter methylation toward aberrant expression of CADM1 in HPV-positive cancer cell lines (Fig. 2A). Hypermethylation was defined as the over 50% methylation of the specific CpG sites. The methylation of the *CADM1* promoter was highly increased in HPV-positive HeLa (71.7%), SiHa (84.8%), and CaSki (95.2%) cells, but was very low in HPV-negative C33A (2.6%) cells (Fig. 2A). Moreover, *CADM1* hypermethylated cervical cancer cells were treated with the DNA methylation inhibitor 5-aza-dC to determine whether methylation of *CADM1* was the reason for its silencing. Treatment with 5-aza-dC induced significant upregulation of gene expression in all highly methylated cell lines (Fig. 2B). These data indicate that hypermethylation of the *CADM1* promoter region might be an active mechanism of silencing of *CADM1* gene expression, and that HPV may play an important role in the loss of *CADM1* gene expression via DNA methylation.

### Demethylation enhances the antiproliferative function of CADM1

**Figure 3:**
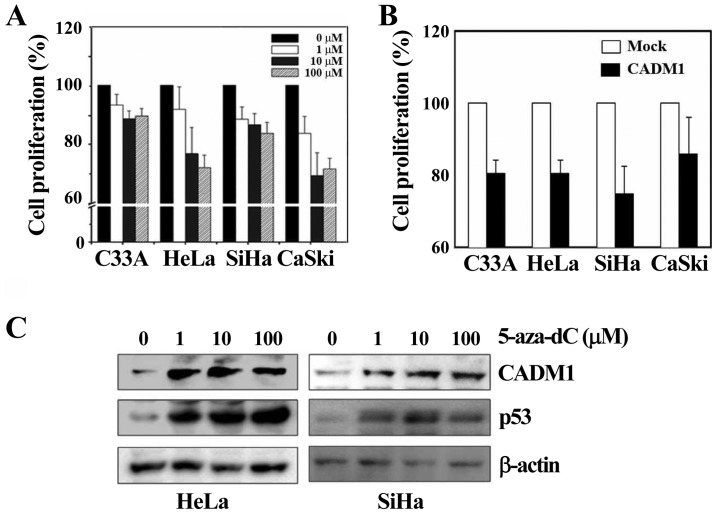
Dysregulation of CADM1 affects cell proliferation in cervical cancer cells. For cell proliferation analysis, 0.5×10^4^ cells/well were incubated with various concentrations of 5-aza-dC (A) or transfected with pcDNA3.1-CADM1 (CADM1) or empty vector (Mock) (B) in a 96-well plate for 68 h, and incubated further with the CCK-8 reagent for 4 h. (C) Western blotting was used to detect the overexpressed CADM1 protein after 5-aza-dC treatment (from 1 to 100 μM). Actin and p53 were used as controls of protein loading and of 5-aza-dC effect, respectively.

To analyze the function of the *CADM1* gene in HPV-induced cervical carcinogenesis, we treated cells with the demethylating reagent 5-aza-dC to reverse the epigenetic silencing dose-dependently, or constructed and transfected a pCDNA3.1-CADM1 expression vector into each of the cervical cancer cell lines. We performed the cell proliferation assay in various cell lines. In the cervical cancer cells (C33A, HeLa, SiHa and CaSki) that were treated with the demethylating agent 5-aza-dC for 72 h, cell proliferation was markedly decreased in all the cell lines tested compared with DMSO-treated samples, and the effect was dose-dependent, although the effect observed in C33A cells (which are negative for HPV infection) was slightly lower (Fig. 3A). In addition, overexpressed CADM1 resulted in a significant decrease in proliferation in all cell lines compared with the control, as shown in Fig. 3B. To determine whether the effect of 5-aza-dC on cell proliferation was induced by restoration of CADM1, we treated HeLa and SiHa cells with 5-aza-dC for 72 h at various concentrations, similar to the protocol used in the proliferation assay. The level of CADM1 was significantly increased in a dose-dependent manner in both cell lines (Fig. 3C). Therefore, these data indicate that 5-aza-dC is effective and that demethylation on the *CADM1* promoter induces CADM1 restoration. It is known that the demethylating agent 5-aza-dC can promote p53 expression by inducing DNA damage (21). Consistently, we also observed that p53 levels were dramatically increased in both cell lines after treatment with 5-aza-dC (Fig. 3C). We further investigated a potential mechanistic link between p53 and CADM1 expression in cervical cancer.

### Effect of p53 on the regulation of CADM1 expression

**Figure 4:**
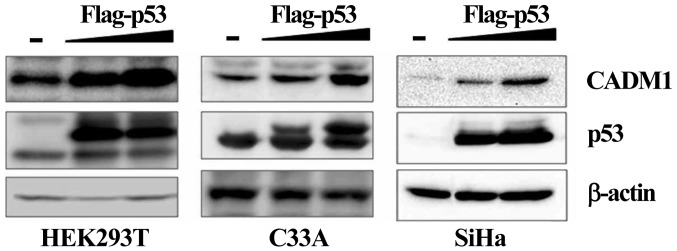
p53 increases CADM1 expression. The CADM1 protein was upregulated by exogenous p53 in a dose-dependent manner in keratinocytes and various cancer cells, including HPV-negative C33A and HPV-positive SiHa cells. After individual cells were transfected with various concentrations of pcDNA3.1-flag-p53 for 48 h, the cells were harvested for western blot analysis of the expression level of CADM1 and exogenous p53 in transfected cells. The expression level of the β-actin protein was considered as the loading control.

**Figure 5:**
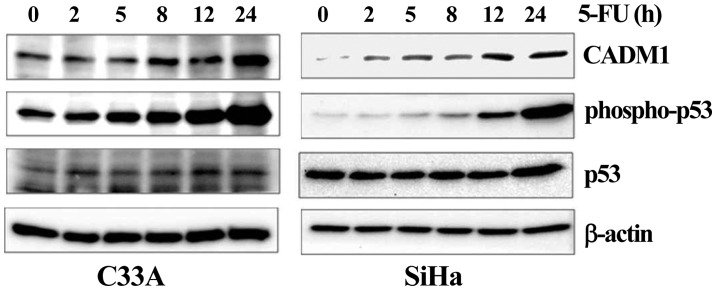
The phosphorylation of p53 by 5-FU upregulated CADM1. The time-dependent effect of 5-FU on the levels of the CADM1, phospho-p53, p53, and β-actin proteins in the C33A and SiHa cervical carcinoma cell lines was determined by western blot analysis. Equal amounts of the cell lysate from each cell line treated with 5-FU (50 μM) at the indicated time were separated on a 10% Tris-glycine gel and immunoblot analysis was performed using specific antibodies, including anti-CADM1, anti-phospho-p53, anti-p53, and anti-β-actin antibodies, respectively.

Degradation of the p53 protein via hr-HPV E6 is a crucial factor for cervical carcinogenesis. In our data (Fig. 3C), treatment with the demethylating agent 5-aza-dC upregulated the p53 protein and induced the CADM1 reactivation by demethylation. To investigate a potential mechanistic link between the upregulation of p53 and CADM1 expression in cervical cancer cells, we selected three cell lines, including HEK293T (non-cervical cancer cell line), C33A, and SiHa (cervical cancer cell line), for transfection with a flag-tagged wild-type p53 expression vector (22) or an empty vector. Successful transfection was confirmed by western blotting, which demonstrated the upregulation of p53 in the transfected cell lines compared with mock transfectants (Fig. 4). Expression of the CADM1 protein was increased by exogenous expression of p53 in a dose-dependent manner in all cells. We further analyzed the effect of endogenous p53 on CADM1 expression by treatment of C33A and SiHa cell lines with 5-fluorouracil (5-FU), an inducer of endogenous p53 activation. Induction of the expression of the phosphorylated p53 active protein was achieved in the selected cell lines after 5-FU treatment. We observed that, in all cell lines, increased phospho-p53 markedly enhanced the expression of CADM1 (Fig. 5). These results demonstrated that the expression of the CADM1 tumor-suppressor gene maybe under the control of p53 and that CADM1 may be another target of the p53 protein.

## Discussion

The cell adhesion molecule 1 *(CADM1)* gene, which is also known as *TSLC1* or *Necl-2*, has been generally investigated as a tumor-suppressor gene in various tumors, including prostate, esophageal, nasopharyngeal, non-small cell lung and cervical cancers (16,18,23–25). Many studies have shown that *CADM1*, as a tumor-suppressor gene, is associated with inhibition of cell proliferation, as well as invasion and induction of apoptosis in various tumor cells, including the non-small cell lung cancer A549 cell line (26), human esophageal carcinoma Eca109 cells (27), and a hepatocellular carcinoma cell line (28). *CADM1* is downregulated in many cancers, frequently via promoter hypermethylation (16,17). However, to date, little is known about the role of the hypermethylation of *CADM1* in cervical cancer.

In our study, we analyzed the protein and mRNA expression of CADM1 in various cervical cancer cell lines, including C33A (HPV-negative), HeLa (HPV18 positive), and SiHa and CaSki (HPV16-positive) cells, and found that CADM1 was significantly downregulated in three HPV-induced cervical cell lines. Moreover, we explored the mechanism underlying the downregulation of CADM1 in each of the cell lines. It is now known that *CADM1* is expressed universally in human tissues and is frequently silenced in a variety of human carcinomas, such as lung, prostate, liver, stomach, pancreatic, and breast carcinoma (26,29). The silencing of *CADM1*, which was first described by Murakami *et al* (30), was explained by loss of heterozygosity on chromosome 11q23 (30) and promoter hypermethylation (16,17). Similarly, the expression of *CADM1* is modulated by genetic and/or epigenetic mechanisms. In our study, we assessed the methylation status of the promoter region of *CADM1* by pyrosequencing. In three HPV-positive cancer cell lines (SiHa, HeLa and CaSki), the average methylation level of *CADM1* was 83.9%. Treatment with 5-aza-dC increased CADM1 expression levels in three cervical carcinoma cell lines that lack the endogenous CADM1 protein, thus confirming that promoter methylation is involved in the silencing of *CADM1* in cervical carcinoma. However, an HPV-negative cervical cancer cell line, C33A, exhibited a methylation level of 2.6%. Based on our data, the methylation levels of C33A (HPV-negative cervical cancer cell line) were significantly lower than those of SiHa, HeLa and CaSki cells (HPV-positive cervical cancer cell lines). Thus, *CADM1* gene silencing might be associated with HPV-mediated malignant transformation.

A previous study showed that new tumor suppressors, such as the secreted frizzled-related protein *(SFRP)* gene, exhibited higher levels of methylation in the HPV-positive than in the HPV-negative group in ovarian cancer, and that the increase in the methylation pattern of the *SFRP1* gene might be due to viral infection and integration into host cells (31). Some authors have also suggested that HPV interferes with the cellular DNA methylation machinery, either to conceal itself or as part of its viral cycle (32). The aberrant promoter methylation of tumor-suppressor genes may result from mistargeted host defenses during viral integration or as a consequence of genomic instability caused by HPV infection. To date, there is no evidence that the silencing of specific genes is linked to the functions of the viral genes expressed after HPV infection. Further studies are needed to determine whether *CADM1* silencing is induced by HPV infection.

Two intracellular oncoproteins, E6 and E7, play important roles in the malignant transformation of HPV-infected cells (4). As the main player in cervical carcinogenesis, E6 activities are mediated by E6-dependent degradation of the tumor-suppressor protein p53. Aberrant regulation of p53 is crucial for cervical carcinogenesis and, most importantly, for the maintenance of the malignant phenotype (4). p53 was activated by 5-aza-dC. 5-Aza-dC treatment is perceived as being DNA damaging and leads to the activation of the G1 checkpoint regulator p53 (21). Our data showed that p53 expression was increased after 5-aza-dC treatment, independent of the presence of HPV. The abnormal expression of p53 target genes is caused by DNA methylation (8–10). Thus, it was of great interest to investigate whether the upregulation of p53 affects the regulation of CADM1. In cancer cell lines with *CADM1* methylation, the induction of CADM1 expression after transfection with p53 was achieved.

We further tested the role of p53 by inducing its endogenous expression using 5-fluorouracil (5-FU). This chemotherapeutic agent is used to treat cancer cells, as it causes irreparable DNA damage, thus inducing these aberrant cells to undergo cell death. It has been reported that 5-FU activates p53 expression and induces p53 target genes during damaged DNA repair (33). Treatment of the cervical cancer cell lines with 5-FU led to the increase of the level of activated p53. Activation of endogenous p53 by 5-FU markedly enhanced the expression of CADM1 in the cell lines with unmethylated *CADM1*. Our data showed that exogenous expression of p53 or increased phosphorylation of p53 regulated CADM1 expression both in the cell line (C33A) with less *CADM1* DNA methylation and in the cell line (SiHa) with a high level of *CADM1* methylation. We presumed that CADM1 expression was regulated by p53, even though we do not know exactly whether p53 binds to the *CADM1* promoter directly or via other mediators. In fact, 5-aza-dC and 5-FU have been considered as a part of a combination therapy with other anticancer agents to treat ovarian, breast, prostate, gastric, lung, pancreatic and colon cancers via the relief of DNA hypermethylation (34) or the growth arrest of cancer cells (35). These results suggest that a therapeutic approach that combines DNA methyltransferase inhibitor drugs that induce the expression or activation of p53 may be a useful strategy for the treatment of cervical cancer. However, the genes that are induced by combination therapy are not limited to *CADM1*, and the involvement of many unknown or known genes that were regulated by the demethylation or *p53* should be considered; further studies are necessary to analyze this issue from different aspects in the context of cervical cancer treatment.

In conclusion, we demonstrated that the inactivation of *CADM1* was associated with its hypermethylation in HPV-induced cervical cancer. To our knowledge, this is first study of the reduction of CADM1 protein levels by epigenetic silencing, and that CADM1 exerts its tumor-suppressive effect via the inhibition of cell proliferation in cervical cancer cell lines. Moreover, CADM1 expression was regulated by exogenously expressed p53 and activated p53 induced by 5-FU treatment. Based on our results, we suggest that *CADM1* methylation is one of the common epigenetic alterations in HPV-infected cervical cancers. In this respect, recovery of *CADM1* gene expression may be a proper target of demethylating agents and p53-regulating drugs in cervical carcinoma. Although much remains to be clarified regarding the aberrant methylation of *CADM1*, further studies should shed light on the mechanism of cancer development, as well as identify more effective cancer therapies.
